# MCU-Dependent mROS Generation Regulates Cell Metabolism and Cell Death Modulated by the AMPK/PGC-1α/SIRT3 Signaling Pathway

**DOI:** 10.3389/fmed.2021.674986

**Published:** 2021-07-09

**Authors:** Yuxin Wang, Xiang Li, Fengchao Zhao

**Affiliations:** Department of Orthopaedic Surgery, The First Affiliated Hospital, Zhejiang University School of Medicine, Hangzhou, China

**Keywords:** mitochondrial calcium uniporter, mitochondrial reactive oxygen species, metabolism, AMPK/PGC-1α/SIRT3, cell death

## Abstract

The mitochondrial calcium uniporter is an intensively investigated calcium channel, and its molecular components, structural features, and encoded genes have long been explored. Further studies have shown that the mitochondrial calcium unidirectional transporter (MCU) is a macromolecular complex related to intracellular and extracellular calcium regulation. Based on the current understanding, the MCU is crucial for maintaining cytosolic Ca^2+^ (cCa^2+^) homeostasis by modulating mitochondrial Ca^2+^ (mCa^2+^) uptake. The elevation of MCU-induced calcium levels is confirmed to be the main cause of mitochondrial reactive oxygen species (mROS) generation, which leads to disordered cellular metabolic patterns and cell death. In particular, in an I/R injury model, cancer cells, and adipocytes, MCU expression is maintained at high levels. As is well accepted, the AMPK/PGC-1α/SIRT3 pathway is believed to have an affinity for mROS formation and energy consumption. Therefore, we identified a link between MCU-related mROS formation and the AMPK/PGC-1α/SIRT3 signaling pathway in controlling cell metabolism and cell death, which may provide a new possibility of targeting the MCU to reverse relevant diseases.

## Introduction

Previous studies have revealed that mitochondria regulate intracellular and extracellular calcium concentrations and signaling and are involved in a series of physiological and pathological processes, including energy metabolism, signaling regulation, smooth muscle contractility, cell proliferation, and cell death. Calcium permeation into cells is driven by the electrochemical gradient between the external space and matrix space (1). Mitochondrial calcium uptake is mediated by a highly selective calcium channel that is localized on the inner mitochondrial membrane called the MCU. Calcium has an extremely high affinity for the MCU, substantially exerting the properties of the MCU (2). Nevertheless, the precise and specific mechanism underlying the function of the MCU remains elusive. Many studies have attempted to illustrate the exact structure and function of the mitochondrial calcium uniporters. As experiments progress, it has been found that the uniporter is a complicated protein complex in humans that consists of four essential elements: MCU [located at the mitochondrial inner transmembrane for ion conduction that is inhibited by ruthenium red (RR) or Ru360] (3), MICU1 and MICU2 (two vital regulators that localize to the intermembrane space as gatekeepers) (4, 5), and EMRE (an essential membrane crossing subunit prompting the entry of calcium) (4). Ca^2+^ participates in various signaling pathways, inducing multiple cellular processes. Nevertheless, calcium uptake by mitochondria is attributed to three main functions: (1) maintenance of cellular metabolic homeostasis between the cytosol and mitochondria, (2) mediation of cCa^2+^ dynamics, and (3) modulation of various cell death pathways of apoptosis and necrosis (6–8). Ca^2+^ enters the mitochondrial matrix under large gradients across the membrane to take part in the tricarboxylic acid (TCA) cycle through dehydrogenase to regulate the generation of ATP, which is crucial to the responses required for energy, such as muscle contraction, exocytosis, biosynthesis, and neuronal signaling (6). Mitochondria attenuate cytosolic calcium through the transportation of calcium when there is a transient influx of Ca^2+^ from intracellular stores and extracellular sources. Moreover, it is also vital for the function under the current conditions of weak intracellular Ca^2+^ buffering (7). Cell death proceeds by constantly increasing mitochondrial free Ca^2+^, resulting in the overloading of Ca^2+^, which ultimately contributes to the intervention of ATP generation and the appearance of mROS (8). Taken together, the MCU functions as a calcium unidirectional transporter and has been demonstrated to affect multiple cell type activities, especially pathological processes. Many experiments have shown that the MCU modulates tumor metastasis (9), ischemia-reperfusion (I/R) injury (10), neuronal apoptosis (11), muscle atrophy (12), and abnormal adipocyte metabolism (13) *via* calcium transition. The AMP-activated protein kinase (AMPK)/peroxisome proliferator-activated receptor gamma coactivator-1α (PGC-1α)/sirtuin-3 (SIRT3) signaling pathway, which is sensitive to the AMP/ATP ratio, is vital for energy metabolism (14). The formation of mROS also reflects the regulation of PGC-1α and SIRT3 on the expression levels of the MCU. In addition, mROS is thought to be the imperative step in triggering apoptosis and necrosis (15). Here, we analyzed the architecture and regulatory factors of MCU and revealed its vital role in the dynamic calcium balance. Moreover, the purpose of this review is to discuss how MCU-dependent mROS generation regulates cell metabolism and cell death, causing pathogenesis. We also explored the relationship between the MCU-mROS axis and the AMPK/PGC-1α/SIRT3 signaling pathway.

## Structure of the Mitochondrial Calcium Uniporter

### The Pore-Forming Subunits of MCU

The uniporter complex has been defined as an inward rectifying current that is universally expressed in all eukaryotes. However, in most fungi, genome sequence analysis indicates that the pore-forming MCU is the only component of the uniporter, which makes it an excellent heterologous expression system to reconstruct the MCU to exhibit uniporter properties in yeast. Based on this, investigators have found that an mCa^2+^ uptake response was evoked after reconstruction, suggesting that MCU is the pore-forming subunit of the uniporter complex (16, 17). Electrophysiological studies have demonstrated that the MCU, an ion channel, exhibits essential selectivity and high affinity for Ca^2+^, but it is sensitive to RR and its analog Ru360 (2). Computational analysis provides a hypothesis of ion channel topologies of the MCU pore-forming domain, which includes four identical subunits composed of two transmembrane helices (TM1 and TM2) separated by a conserved linker. The linker, facing the intermembrane space, is a short stretch of amino acids and contains a motif called “DIME (Asp-Ile-Met-Glu)”. In addition, the structure of the MCU comprises two coiled coils domain (CCD) and an N-terminal domain (NTD) (3, 18). In recent years, researchers have applied cryo-EM to further study the precise architecture of MCU. The overall structure is in accordance with the previously predicted hypothesis. MCU displays an exactly tetrameric architecture by means of EM under both high and low concentrations of calcium. TM1 and CC1 form a long and continuous helix at the periphery of the channel, while the TM2 helices line the central symmetry axis. There is a short helix that is defined as a junctional helix (JH), situated almost perpendicular to TM1. JH forms a junction between TM2 and CC2. In the CCD, CC1 and CC2 form a dimeric coiled coil, resulting in four dimeric coiled coils in the tetramer. Following the coiled-coil domain, the NTD, comprising six β-strands (β1–β6) and two α-helices (α1 and α2) that form the central core with two highly conserved leucine-rich loops (19), is directly connected to CC1 (20). The structure of the NTD forms the MCU oligomers, and deletion of the NTD results in a rapid decline in mCa^2+^ with complete cCa^2+^ dynamics (19). A residue of the NTD called the MCU-regulating acidic patch (MRAP) binds divalent cations to autoregulate Ca^2+^ uptake by matrix Ca^2+^ and Mg^2+^, occupying the MRAP domain, similar to a feedback mechanism (21). The strictly conserved sequence motif existing in all MCU homologs has been demonstrated to shape the selectivity filter of Ca^2+^. This sequence motif is located in the N-terminal region of TM2, with the carboxylate side chains of conserved acidic residues Asp and Glu from each protomer pointed into the central symmetry axis, forming two acidic rings along the channel pore. Asp is located at the intermembrane end of TM2 and its carboxylate ring coordinates hydrated Ca^2+^, while the diameter of the Glu carboxylate ring is too small for hydrated Ca^2+^ to pass through, suggesting that only dehydrated Ca^2+^ is allowed to pass through (17, 20, 22). Consequently, we have enough evidence to deduce that the Glu carboxylate ring is unsubstitutable for selective permeation.

### The Regulatory Subunits of MCU

It is generally accepted that the primary regulatory subunits centered around Ca^2+^ channel proteins are MICU1, MICU2, MCUR1, MCUb, and EMRE. MICU1, co-occurring with MCU in mammals but absent from most fungi, is a peripheral membrane protein with two EF-hand motifs that functions as a calcium-sensing regulator (23). MICU1 interacts with MCU through the D-ring of the DIME motif to constitute the conserved unit of a eukaryotic uniporter. It is not until cCa^2+^ rises above 3 μM that the gatekeeper MICU1 will activate the MCU channel by dissociating from MCU, making it adopt an open confirmation (24). To prove this hypothesis, genetic deletion of MICU1 was performed, and the results complemented the assumption that mCa^2+^ increase, even at low cCa^2+^, simultaneously caused mCa^2+^ overload (25). Moreover, when MICU1 was knocked down, the sensitivity of RR/Ru360 to MCU increased, while calcium uptake remarkably decreased, demonstrating that MICU1 likely competes with RR/Ru360 at the site of the pore-forming domain (5, 26).

MICU2 is a paralog of MICU1 and shares a similar function as a gatekeeper that assists MCU in taking up calcium from the cytoplasm. Silencing MICU2 by RNAi revealed a reduced rate of mCa^2+^ uptake, signifying that MICU2 is dispensable for mCa^2+^ uptake (27). Importantly, utilizing the transcription activator-like effector nuclease (TALEN) technology to knock out MICU1 and MICU2 in HEK293 T cells resulted in a steep Ca^2+^ uptake by MICU1 KO mitochondria due to the loss of gatekeeping. MICU2 KO mitochondria exhibited a decreased rate of Ca^2+^ influx (28). It follows that MICU2 may have a positive effect on the cooperation with MICU1 to achieve the function of gatekeeping. In addition to MICU1, heterooligomers of MICU1 and MICU2 also interact with MCU to control the gating and cooperative activation of the uniporter. However, the respective function and mechanism of each subunit in mediating the activity of the uniporter is complicated in the mammalian system because of differences ranging from the degree of gene silencing, tissue-specific protein composition (29, 30) from stoichiometry, and compensatory remodeling of the channel (25, 31).

In contrast, MCU regulator 1 (MCUR1), a positive regulator of the channel, exerted a blunt MCU current when the transmembrane voltage was clamped, indicating that MCUR1 acts directly on MCU to promote Ca^2+^ entry into the mitochondria instead of the reduced electrochemical driving force (32). Another laboratory finding showed that MCUR1 is an assembly factor of complex IV cytochrome oxidase, leading to an impaired oxidative phosphorylation in the absence of MCUR1 that is certainly a decreased driving force that decreases calcium uptake (33).

MCU proteome analysis revealed that EMRE, a 10-kilodalton protein with an EF hand domain, is ubiquitously expressed in all mammalian mitochondria. Experiments have demonstrated that a knockout of EMRE specifically decreases the uptake of calcium, while the appearance and proliferation of cells is not affected. Moreover, the abundance of other uniplex proteins and their mitochondrial localization also remain. What has changed is the association of MCU with MICU1 and MICU2. Hence, we can link the function of EMRE and MICU to calcium-sensing activity (4). Moreover, it has been proposed that EMRE senses the matrix calcium concentration with the acidic patch at its carboxy terminal to regulate MCU activity (34). However, in the study by Tsai et al., the interaction of MICU1 with MCU in the absence of EMRE was also displayed by coimmunoprecipitation (35).

**Figure 1:**
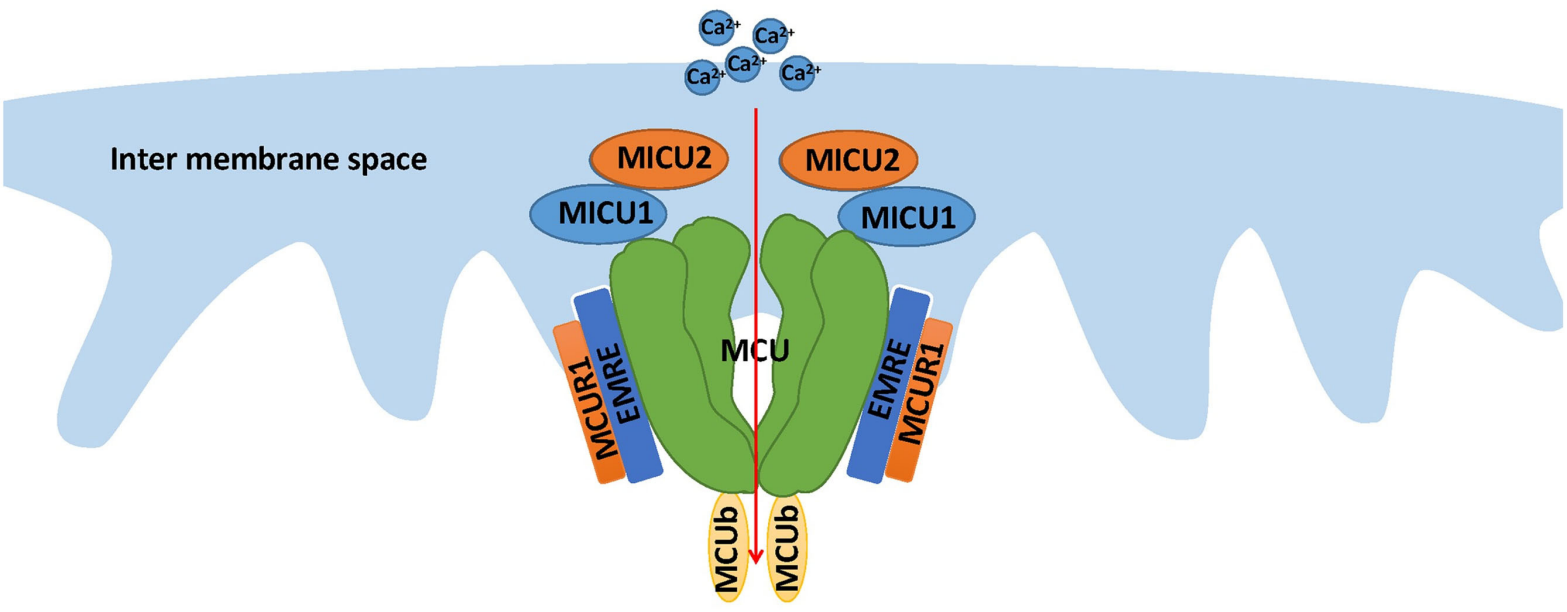
The structure of the MCU complex. This compound, localized at the inner membrane of the mitochondria, consists of the pore-forming subunit and regulator subunits, including MICU1, MICU2, EMRE, MCUR1, and MCUb. The most important function of MCU is mediating mitochondrial calcium uptake.

Recently, MCUb, a paralog of the pore-forming subunit MCU, was investigated to determine whether it has a prominent influence on Ca^2+^ permeation. MCUb is conserved across all vertebrates and absent in species of plants, kinetoplastids, Nematoda, and Arthropoda, where MCU is present (18). The results of the present experiments suggest that MCUb is an important continuance of the uniplex and has a negative effect on Ca^2+^ uptake. MCUb dislocates the position of MCU to disconnect MCU from MICU1 and MICU2 to alter channel gating. Meanwhile, the presence of MCU and the expression of channel regulators MICU1 and MICU2 rapidly increases after MCUb depletion (36) (Figure 1).

## Modulation of MCU Expression

**Figure 2:**
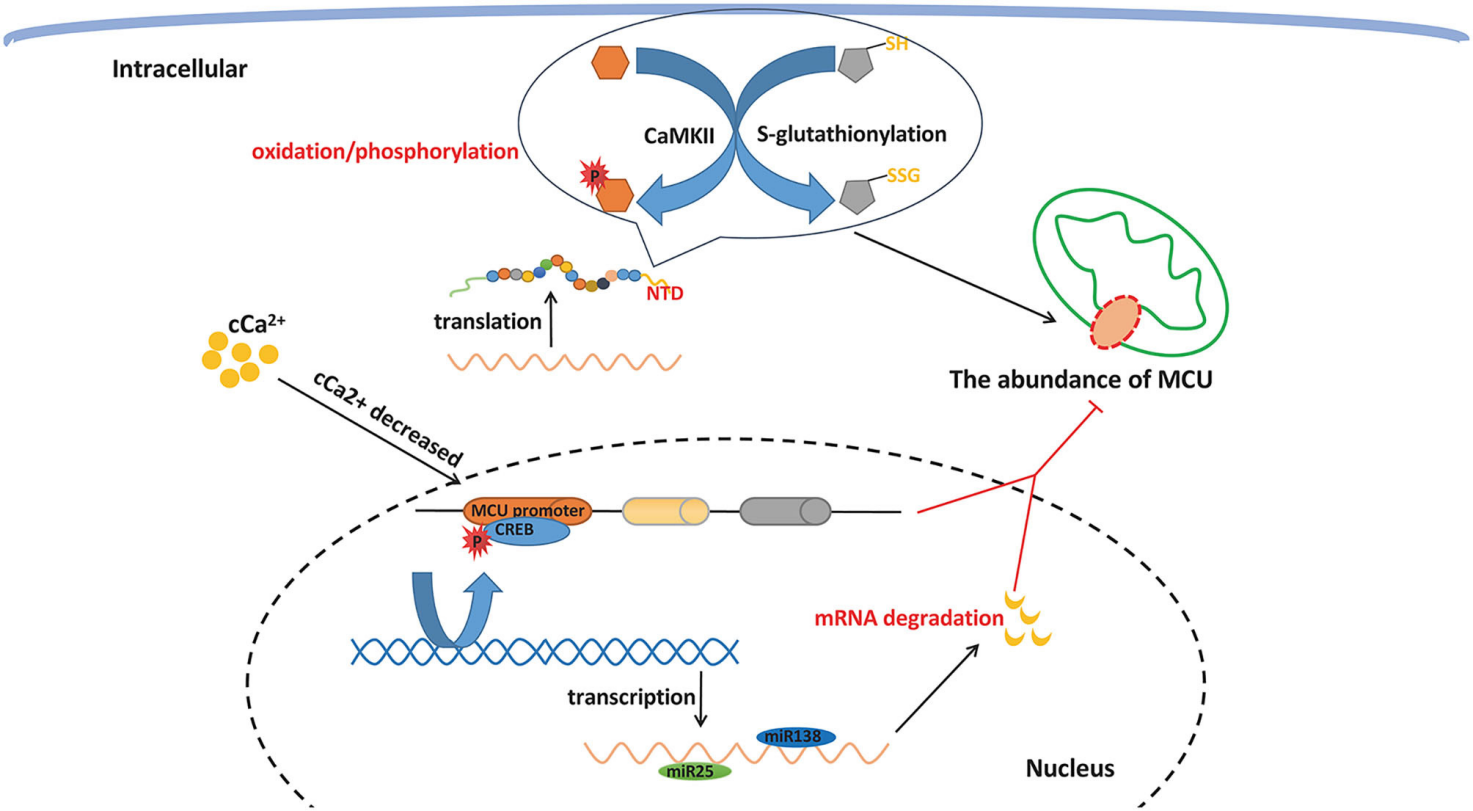
MCU expression regulation. MCU protein generation can be impacted at several molecular levels, including transcription, post transcriptional modification, translation, and post translational modification, which can remarkably change the interplay between cCa^2+^ and mCa^2+^ *via* influencing the expression level of protein.

As demonstrated in experiments, cCa^2+^ signaling is influenced by both high and low cytosolic calcium concentrations (26, 36). MCU, a core ion channel transmitting Ca^2+^, may be an effective target to interrupt Ca^2+^ homeostasis and subsequently be involved in various pathological processes. In line with this finding, regulating the processes associated with MCU protein generation, including transcription, posttranscriptional modification, translation, and posttranslational modification (PTM), remarkably influence the expression level of proteins to change the interplay between cCa^2+^ and mCa^2+^. The Ca^2+^ regulated transcription factor CREB (cyclic adenosine monophosphate response element) binds to the MCU promoter. When cCa^2+^ decreases, phosphorylation of CREB is instantly initiated to alter transcription followed by the downregulation of MCU abundance and the decrease in mCa^2+^ (37). At the posttranscriptional level, microRNAs (miRNAs) are non-coding nucleotides and can modulate gene expression by combining with specific miRNAs to degrade target miRNAs or restrain translation (38). There have been valid findings linking miRNA-associated MCU expression with cell survival. The downregulation of MCU RNA and protein by miR-25 has been shown to protect tumor cells from cell death in colon cancer (39). Furthermore, subsequent studies utilized anti-miR25 and anti-miR138 in pulmonary arterial hypertension (PAH) patients whose MCU expression is downregulated, and the final conclusion revealed the predictable overexpression of the MCU protein (40). PTMs are composed of two predominant forms, namely, oxidation and phosphorylation (41). Previous studies confirmed the findings about the PTMs of MCU. In the NTD of MCU, there are conserved cysteines, which carry S-glutathionylation under oxidative stress to reconstruct NTD confirmation, which accelerates the sustained activity of MCU and a high rate of calcium uptake. In addition, phosphorylation is proposed to occur in the MCU PTM at sites S57 and S92 of the NTD in the presence of calmodulin kinase II (CaMKII). The implication from further studies revealed facilitated MCU current compared to the control, which inhibited the expression of CaMKII (19). These findings highlight the significance of regulating MCU expression and propose a novel route to interpose cell death and metabolic homeostasis (Figure 2).

## MCU Is Involved in the Process of Metabolic Homeostasis AND Cell Death Via MROS

### The Generation of mROS Under Elevated Mitochondrial Ca^2+^ Levels Induced by MCU

Mitochondria function in buffering the concentration of intracellular calcium. Mitochondria isolated from rat kidneys in the 1960s showed a potent ability to uptake calcium even at a low temperature (42). As studies have indicated, MCU, which displays a high affinity for calcium, was revealed to be the Ca^2+^ transportation protein localized in the IMM. Expression levels of MCU and calcium current were also found to be different in various tissues, including the heart, skeletal muscle, liver, and neurons. Fieni et al. (17) performed studies that provided evidence of a large density of MCU in neonatal cardiac tissue through patch-clamp experiments. The biochemical gel shift assay provided evidence that MCU is the only modality that senses mROS. When inflammation and oxidative stress increase mROS, modification of channels by mROS causes mCa^2+^ overload-mediated cell death. In turn, with changes in the extracellular microenvironment and the stimulation of hypoxia, the elevated mCa^2+^ taken up by MCU interrupts energy metabolism, causing the generation of mROS.

**Figure 3:**
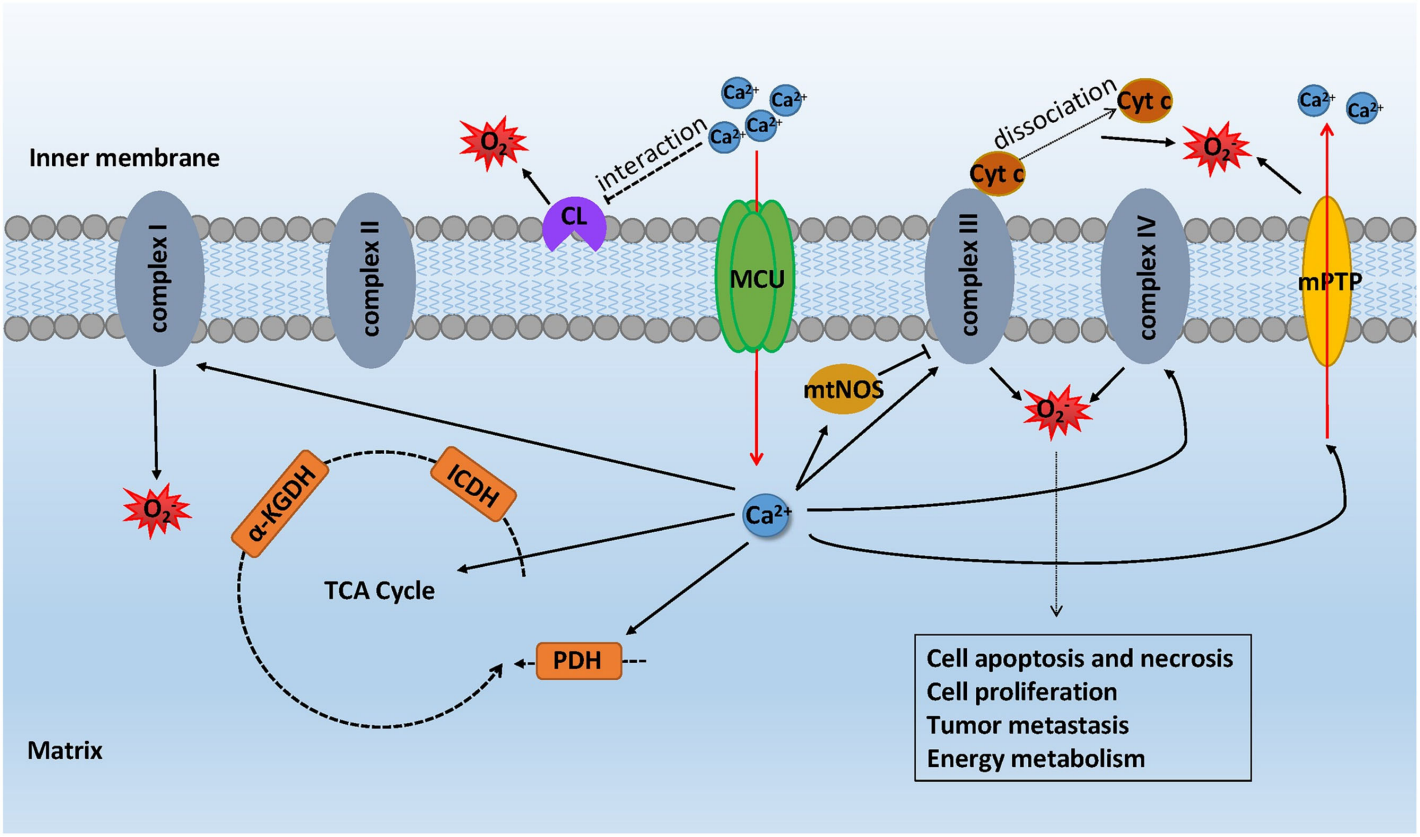
mROS formation mediated by MCU-dependent calcium uptake. With the stimulation of oxidative stress, the elevated mCa^2+^ took up by MCU interrupts energy metabolism causing the generation of mROS while the mROS formation can impact the cell metabolic function in turn. mCa^2+^ increases the activity of glycerol phosphate dehydrogenase (GPDH), pyruvate dehydrogenase (PDH), isocitrate dehydrogenase (ICDH), and α-ketoglutarate dehydrogenase (α-KGDH) in the TCA cycle, thus promoting mROS production. mtNOS, activated by mCa^2+^, hampers the conduction of electron, making complexes I and III generate mROS. mPTP opening results in the rupture of the outer membrane and thus causing the release of cytochrome c. Overloaded mCa^2+^ has an effect on Ca^2+^-CL binding, inducing the reorganization of membrane components, which causes the high-rate generation of mROS at the ubiquinone level.

There are methods to induce the production of mROS with an increased mCa^2+^: (i) dehydrogenases that participate in the tricarboxylic acid cycle, including glycerol phosphate dehydrogenase (GPDH), pyruvate dehydrogenase (PDH), isocitrate dehydrogenase (ICDH), and α-ketoglutarate dehydrogenase (α-KGDH), are sensitive to mCa^2+^ (43). They cannot completely work without the existence of mCa^2+^. In contrast, the data show that an elevated mCa^2+^ stimulates the activity of these dehydrogenases and even the conductance of complexes I, III, IV, and V in OXPHOS (44). A large amount of O_2_ accelerates cellular metabolism, increasing the generation of mROS, which is a byproduct of the electron transport chain, although a low rate of O_2_ consumption has also been discovered to promote ROS production (45, 46). (ii) mtNOS, a constitutive nitric-oxide synthase (NOS) isoform, requires calcium to activate its function. Under high concentrations of mCa^2+^, mtNOS tends to decrease oxygen consumption *via* the competitive binding of nitric oxide to cytochrome oxidase. Reduced O_2_ consumption and the competitive binding of cytochrome oxidase hamper the conduction of electrons, which gives rise to a higher tendency of inducing complexes I and III to generate mROS (47). (iii) Opening of the mitochondrial permeability transition pore (mPTP) induced by MCU-dependent calcium overloading abruptly admits all solutes of molecular weight up to approximately 1,500 Da to permeate into the mitochondrial matrix, which results in mitochondrial depolarization, mitochondrial swelling, and rupture of the outer membrane, causing the release of cytochrome c (48–50). Intriguingly, opening of the mPTP can change the ionic strength within the intermembrane space, destroying the electrostatic interaction between cytochrome c and cardiolipin. The release of cytochrome c is triggered by the disruption of interaction, which blocks the activity of complex III and thus intercepts electron flow to enhance mROS production (46, 51). (iv) Studies have supported the hypothesis that Ca^2+^-cardiolipin (CL) interactions with cardiolipin headgroups mainly contribute to multiple membrane dysfunction. With regard to overloaded mCa^2+^, Ca^2+^-CL binding induces a sequence of chemical, dynamic events that favor the reorganization of membrane components, which causes the high rate generation of mROS at the ubiquinone level (52) (Figure 3).

### mROS Dysregulation Induced by MCU-Dependent Calcium Influx Causes Metabolic Disorders and Cell Death

As mitochondrial calcium is important for modulating mitochondrial activity, the change in MCU expression causes aberrant mCa^2+^ levels, which provokes the increasing production of mROS, as previously described. Obesity or age-related whitening and loss of brown adipose tissue (BAT) contribute to systemic metabolic dysfunction and the facilitation of energy storage, which has been demonstrated in a prior study (53). Wright et al. (13) reported that expression levels of the MCU complex were increased in mouse and human adipose tissue during obesity. In insulin-resistant (IR) adipocytes, MCU and its relevant components have been observed to be upregulated together with an increased mCa^2+^ uptake. Experiments have been performed on human IR adipocytes both *in vivo* and *in vitro*, which signified slightly different outcomes. The expression of MCU and MICU1 increased, while the other components, such as MICU*2*, were unaltered *in vitro*. In contrast, MCU, MICU1, and MICU2 all increased in the *in vivo* experiments. In conclusion, the level of MCU was upregulated both *in vivo* and *in vitro*. For this reason, the study further explored the relationship between inflammatory factors and the MCU. The data revealed that ad-MCU caused a decreased release of TNF-α and IL-6, indicating that markedly decreasing the MCU-induced calcium uptake remarkably influences inflammatory cytokine release. These results indicate a possible role for MCU in cell metabolism in adipocytes. However, the precise mechanism remains to be fully understood. In addition, several previous studies have reported the enhanced sensitivity of insulin in adipocytes under decreased intracellular calcium concentrations (54). Given the crucial modulatory function of calcium, with ER-mitochondria contact, ER calcium stores can be rapidly transferred to the mitochondria upon demand *via* MCU transmission (55). Gao et al. (56) performed experiments and discovered that in high-fat diet (HFD)-fed mice, total ROS levels assessed by dihydroethidium in frozen slices of BAT were remarkable increase. Meanwhile, oxygen consumption, carbon dioxide generation, energy expenditure (EE), and respiratory exchange ratio (RER) were also found to be reduced in HFD-fed mice. Moreover, the author observed a significantly elevated expression of the MCU and gatekeeper MICU1, but MCUb and MICU2 were not affected. Interestingly, MCU knockout by si-MCU completely blocked the uptake of mitochondrial calcium and dramatically reduced the excessive production of mROS, improving mitochondrial metabolic activity. In a study of idiopathic pulmonary fibrosis (IPF) subjects, researchers found that PGC-1α, which increases the enzymatic capacity for fatty acid oxidation (FAO) and abolishes glycolysis, was augmented in macrophages, whereas the effects of PGC-1α were diminished in a dominant-negative MCU model (57–59). These results may indicate the dysfunction of MCU, causing a disrupted aerobic respiratory function and an abnormal metabolism.

Intracellular calcium, acting as a second messenger, ubiquitously participates in all kinds of cellular biological events. Because of the importance of Ca^2+^ in signaling pathways, cCa^2+^ dynamics are strictly controlled. A significant quantity of data indicates that a small amount of calcium entering the mitochondria is beneficial to metabolic homeostasis, while a large amount of calcium is thought to induce cell death (60). Liao et al. (10, 43) studied the role of MCU in oxidative-induced cell death. HeLa cells treated with H_2_O_2_ generate massive mROS, arousing apoptosis, which was significantly inhibited by MCU overexpression. It was also discovered that MCU knockdown markedly suppressed mROS-induced apoptosis. To further confirm the importance of mCa^2+^, the researchers induced mutations, MCU_D260A_ and MCU_E263A_, to make it lose the ability to absorb Ca^2+^. The expression of MCU_D260A_ and MCU_E263A_ was unable to achieve oxidative stress-induced apoptosis when treated with H_2_O_2_. Therefore, the mCa^2+^ uptake activity of MCU is essential for facilitating mROS-induced cell death. Accordingly, numerous cell death models, such as cardiac I/R injury and neuronal excitotoxicity, have validated the evidence of its negative effect in the I/R injury mouse model (10, 43). The abundance of MCU protein has been demonstrated to be increased, followed by a high concentration of mCa^2+^ in I/R injury. mROS production occurs after blood reenters into ischemic tissue, causing reperfusion injury. Sharply elevated mROS leads to the opening of the mPTP, which eventually provokes apoptosis and autophagy (60, 61). Proapoptotic factors, such as cytochrome c, are released into the cytosol by ruptured mitochondria, which can upregulate Bax and the Bax/Bcl-2 ratio and activate caspases and other apoptotic events, ultimately culminating in cell death (61). Therefore, the MCU-mROS-mPTP axis primarily regulates the cell death pathway. In addition, oxidative stress-stimulated formation of mROS alters the dynamic balance of mitochondrial fission and fusion under high levels of mCa^2+^. Such ruptured mitochondria trigger a sequence of cellular apoptosis events (62). Dynamin-related protein 1 (Drp1) and mitochondrial LC3II expression indicate the occurrence of fission and mitophagy, respectively (63). A previous investigation confirmed that MCU inhibition alle*via*tes Drp1 accumulation and enhances mitochondrial LC3II expression. Additionally, atrophy type 1 (OPA1) expression, which is supposed to dominate mitochondrial fusion and is suppressed in I/R injury models, was recovered when MCU was repressed. Calpains, which are calcium-dependent thiol-proteases, are activated during I/R injury by calcium overload to phosphorylate Drp1. Nevertheless, the downregulation of OPA1 by siRNA transfection surprisingly abrogates the protective effects of calpain suppression on mitochondrial fission/fusion and mitophagy, indicating that MCU repression restrains calpain/OPA1-mediated mitochondrial fusion/mitophagy inhibition to shrink the myocardial infarction size and reduce levels of apoptosis (64).

In conclusion, high-level expression of MCU-induced mCa^2+^ overloading results in a disordered mitochondrial metabolism and cell death, which primarily depends on the generation of mROS.

## The Role of MCU in Relevant Diseases

As a high level of calcium is reported to change the pattern of cellular energy metabolism from glycolysis to fatty acid oxidation and produce excessive mROS *via* MCU overexpression, the development of various diseases is closely associated with it (13).

ROS production has been well demonstrated to play a crucial role in many types of cancer (65, 66). The primary source of ROS is the mitochondrial electron transport chain (ETC) in most mammalian cells. The generation of ROS, which is mostly dependent on NADH/NAD, is correlated with cellular metabolism. ROS-high tumor sphere (RH-TS) cells were found to have higher intramitochondrial superoxide levels than normal cells. The intramitochondrial superoxide level measured by flow cytometry suggested that endogenous ROS in RH-TS cells were largely generated in mitochondria. With the generation of mROS, the biosynthesis of fatty acids in RH-TS cells was reduced, while the breakdown of fatty acids increased (67). Liu et al. (68) reported that MCU is instrumental for the growth of colorectal cancer (CRC). RT-qPCR and Western blotting analysis showed that the expression levels of both the MCU protein and mRNA were markedly upregulated in the majority of malignant colorectal tissue compared to the adjacent normal tissue. Immunohistochemical and Kaplan-Meier analyses also validated these results. More importantly, studies indicated that treatment with H_2_O_2_ and ROS scavengers reversed the effects of MCU knockdown and MCU overexpression *in vivo*. Taken together, these findings reveal that MCU-mediated calcium uptake stimulates mitochondrial biogenesis to expedite CRC growth *via* excessive ROS generation. Similarly, in hepatocellular carcinoma (HCC) cells, the upregulation of MCU and the downregulation of MICU1 led to an increased basal mCa^2+^ compared to the control cells. It is well known that the electron transport chain is substantially strengthened by increased calcium entering the mitochondria, generating additional mROS. In particular, mCa^2+^ uptake increases NAD^+^ conversion into NADH and downregulates the deacetylase activity of SIRT3, inactivating SOD2, which subsequently loses the capacity to eliminate mROS. As a result, HCC proliferation and cell migration and invasion are enhanced *via* ROS in response to high expression levels of MCU (69). Beyond these, Arvizo et al. (35) probed novel roles of MCU in ovarian cancer. Tosatto et al. (70) found that triple-negative breast cancer (TNBC), the most aggressive breast carcinoma subtype, is also influenced by MCU in terms of tumor size, lung metastasis, and lymph node infiltration by creating an MCU deletion in an MDA-MB-231 cell model with CRISPR/Cas9 nuclease RNA-guided genome editing technology.

In addition, MCU is involved in cardiac I/R injury and neurodegenerative disease. The cardiac microvasculature is particularly susceptible to the deleterious effects of I/R injury, which are mediated by dysregulated intracellular calcium. Li et al. (71) reported that sarco/endoplasmic reticulum Ca^2+^-ATPase (SERCA) recycles calcium from the cytosol back to the endoplasmic reticulum, preventing I/R-induced luminal stenosis, vascular wall edema, endothelial barrier integrity, and erythrocyte morphological changes. Furthermore, they found that SERCA overexpression suppressed MCU expression and simultaneously attenuated intracellular calcium overloading. Applying a calcium activator or using an MCU agonist remarkably induced endothelial necroptosis *in vitro* and abolished the protective effects of SERCA overexpression in a reperfused heart tissue *in vivo*. Signal transducer and activator of transcription 3 (STAT3) in intermittent hypobaric hypoxia (IHH) has been demonstrated to convey cardio-protection against I/R injury. Wu et al. (72) further studied its mechanism in a post-ischemic myocardial cell model. These cardioprotective effects were abrogated by the STAT3 inhibitor AG490, which opens the MCU. Colocalization of STAT3 and MCU was also observed in rat I/R cardiomyocytes. The results of this study ultimately revealed that activated STAT3 interacts with the NTD of MCU to alle*via*te mitochondrial calcium overload, protecting against cardiac I/R. Enlightened by the examination of MCU in I/R injury of cardiomyocytes, MCU-mediated neuron apoptosis and necrosis, which crucially account for neurodegenerative and neural ischemic disease, have been universally probed to identify a novel therapeutic target. Veronica et al. (73) have investigated whether that mCa^2+^ overload is sufficient to decide the neural cell fate by overexpressing MCU both in an exosomatic model of mouse primary cortical neurons and an endosomatic model of injecting MCU-coding adenoviral particles into the mouse brain cortex. In a rat cerebral ischemia model, MCU was observed to be directly phosphorylated by activated proline-rich tyrosine kinase 2 (Pyk2) and aroused Ca^2+^ overloading and mROS accumulation, thus leading to neuronal apoptosis and ischemic stroke. Inhibiting the phosphorylation of MCU with Pyk2 antagonist can prevent mCa^2+^ overload, mitochondrial injury, proapoptotic protein release, and cell death (74). In addition, Maria et al. (75) and Smijin et al. (76) reported the relevance of hereditary spastic paraplegia (HSP7) and Parkinson's disease (PD) to MCU function, respectively. The researchers found that the mitochondrial matrix ATPase associated with diverse cellular activities (m-AAA) protease plays an important role in regulating the function of mitochondria. Protein synthesis and respiration in mitochondria are damaged after inhibiting m-AAA (77). In particular, m-AAA depletion impairs MCU assembly, leading to degradation of the EMRE subunit, which induces the massive entry of mCa^2+^ into the Purkinje cells and ultimately evokes HSP7. Dopaminergic neurons with hybridization and immunohistochemical labels were rescued after the deletion of MCU in the model of PD (76, 78). Alzheimer's disease (AD) is a progressive neurodegenerative disorder with cognitive dysfunction in an individual. The experiments found the blockade of MCU exhibits neuroprotective activity of AD animal models, which was induced by intracerebroventricular injection of streptozotocin (ICV-STZ). In addition, the experimental rats were treated with the MCU blocker, RR. The results indicated that RR attenuated ICV-STZ induced memory-related behavioral abnormalities and decreased level of acetylcholine and choline acetyltransferase activity. The percentage of apoptotic cells in ICV-STZ challenged rat brain regions was also alle*via*ted by RR (79).

## The Relationship Between the MCU-MROS AXIS And the AMPK/PGC-1α/SIRT3 Signaling Pathway

As AMPK/PGC-1α is an intracellular energy sensor and modulator, it is activated in response to energy depletion and high demands for energy supply. SIRT3 that served as the downstream target of AMPK-PGC-1α signaling plays a key role in the regulation of mitochondrial biogenesis and oxidative stress. Most studies have proved that this pathway is related to ischemia-reperfusion injury, neurodegenerative diseases, and abnormal glucose metabolism in diabetes (80, 81). It has a high degree of overlap with the diseases involved in the MCU-mROS axis.

Previous works have provided evidence that decreased SIRT3 expression is the primary feature of BAT whitening. Unlike SIRT1, which primarily exists in the nucleus, SIRT3 preferentially localizes in the mitochondria (82). SIRT3 knockout (KO) or si-Sirt3 significantly promoted MCU expression *via* increased acetylation of histone lysine 27 on the MCU promoter. Moreover, with a decrease in H3K27ac, MCU expression was remarkably reduced. These conspicuous phenomena demonstrate that acetylation of H3K27 plays a key role in the histone modification status to control the transcription of MCU (56). It is well accepted that SIRT3 is an NAD^+^-dependent mitochondrial deacetylase that deacetylates SOD2 to eliminate the formation of mROS. MCU-mediated mCa^2+^ uptake downregulates the NAD^+^/NADH ratio, which diminishes SOD2 deacetylation activity. Furthermore, the activity of SIRT3 and SOD2 drastically reversed MCU overexpression-induced HCC cell migration and invasion (69). Interestingly, SIRT3 not only modulates the metabolic enzymes of mitochondria but also directly regulates mitochondrial morphology dynamics by targeting OPA1 during stress (83). Additionally, Kong et al. (84) reported that SIRT3 was required for PGC-1α-essential induction of ROS-detoxifying enzymes and several constituents of the respiratory chain, such as glutathione peroxidase-1, ATP synthase 5c, and cytochrome c. The data show that the significantly increased deacetylase activity of SIRT3 is consistent with MCU knockdown, whereas MCU overexpression induces the reverse condition (69). In mouse adipose tissue, knockout of SIRT3 increased mitochondrial oxidative stress, resulting in accelerated obesity and metabolic syndrome (85). Capsaicin was proven to be significant in increasing BAT activity and aerobic respiratory function. SIRT3 mediated the promotional effects of capsaicin on BAT metabolism and ROS generation by enhancing MCU activity. The inhibitory effect of capsaicin on driving mitochondrial calcium overload in BAT was impaired after SIRT3 KO. Likewise, mitochondrial ROS were elevated in cells treated with si-Sirt3, compared to WT cells. Gao et al. (56) indicated that SIRT3 alle*via*tes mCa^2+^ overload by inhibiting MCU expression. Nevertheless, both overexpression and knockdown of MCU failed to alter the expression of SIRT3, which indicates that MCU is a downstream target of SIRT3. In addition, researchers constructed a cardiac I/R injury model *via* coronary artery ligation and reperfusion, which showed that mice lacking cyclophilin D exhibited a reduced infarct size. Hafner et al. (86) discovered that SIRT3 deacetylates cyclophilin D on lysine 166 to interrupt the opening of the mPTP. Cardiac myocytes from mice lacking SIRT3 exhibit an increased mitochondrial swelling with age due to ascending mPTP opening, eventually contributing to cell death. Experimental results demonstrate that in both muscle cells and hepatocytes, PGC-1α strongly stimulated mouse SIRT3 gene expression by activating the SIRT3 promoter, indicating that SIRT3 works as a downstream target gene of PGC-1α (84). Activation of AMPK is a sensor of energy metabolism that increases mitochondrial biogenesis, glucose uptake, and fatty acid oxidation by upregulating gene expression in these pathways. It has also been found that activation of AMPK inevitably induces PGC-1α gene expression. In turn, the effects of AMPK on energy metabolism are almost entirely dependent on the PGC-1α protein. AMPK directly phosphorylates PGC-1α to initiate downstream cellular events (87). However, experiments have shown that loss of SIRT3 impacts the phosphorylation of AMPK and PGC-1α expression in skeletal muscle. Accordingly, SIRT3 upregulation strengthened the downstream activation of PGC-1α *via* a higher phosphorylation level of AMPK compared to the control group (88). These results suggest that SIRT3 and AMPK form a positive-feedback loop, acting as a link between the energy metabolism of cells and mitochondrial function.

**Figure 4:**
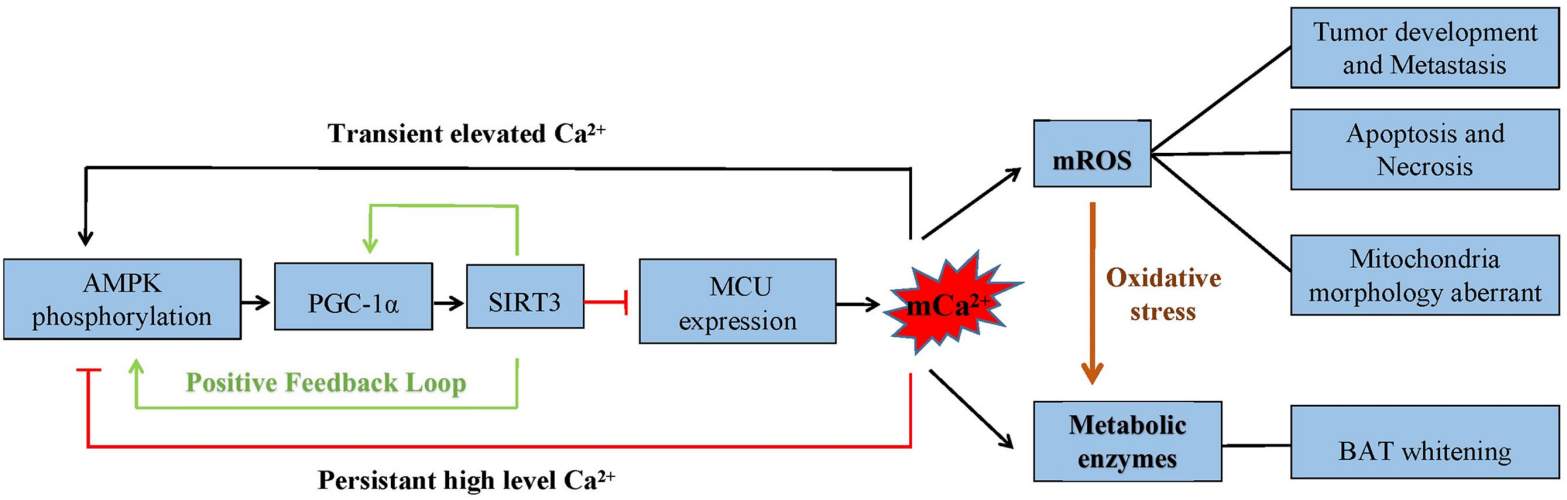
The AMPK/PGC-1α/SIRT3 signaling pathway inhibits the expression of MCU. The capability of mCa^2+^ uptake is decreased, which inhibits mROS generation. Cell metabolism, apoptosis, and necrosis are significantly affected by mCa^2+^-dependent mROS production. Also, the crosstalk between AMPK/PGC-1α and SIRT3 forms a positive feedback loop. The phosphorylation of AMPK is found to be different under a transient and persistent elevation of calcium.

Increasing studies have investigated whether mitochondria act as cCa^2+^ buffers. The rapid accumulation of mCa^2+^ occurs when cCa^2+^ increases. Consistently, different patterns of mCa^2+^ elevation cause contrary results. Investigations have observed that transient mCa^2+^ accelerates mitochondrial bioenergetics and activates the energy metabolism cycle, while calcium overload increases mROS levels and evokes mitophagy (89–91). Analogously, with a transiently elevated cCa^2+^, AMPK phosphorylation is significantly activated, but AMPK activity is blocked by sustained high intracellular Ca^2+^ levels. There may be a close connection between calcium-modulated mitochondrial bioenergetics and the AMPK activated state (92, 93). This phenomenon may provide a defense mechanism to prevent cells from undergoing apoptosis and autophagy under acute stress (Figure 4).

## Perspective

Calcium has been reported to change the pattern of cellular energy metabolism from glycolysis to fatty acid oxidation, which is commonly presumed to affect cellular function and even pathogenesis (13). Mitochondria have consistently played an irreplaceable role in maintaining Ca^2+^ homeostasis, which is largely dependent on the MCU. With the increase in the understanding of MCU, a spectrum of experiments has been performed to reveal the effects of MCU in a myriad of physiological and pathological processes. However, depending on the cell type, an abnormal expression of MCU has distinct effects (9). In neurodegeneration, I/R injury of cardiac and neurologic tissue, skeletal muscle metabolism, cancer cell metastasis, and cell death, mitochondria control Ca^2+^ signaling as a common final pathway in controlling mROS production and organelle structure. Although the AMPK/PGC-1α/SIRT3 pathway has been proven to be crucial in governing the cellular metabolic state and cell death, integral mitochondrial calcium signaling networks remain unknown but has become vital for our understanding of a series of therapeutic targets in various diseases.

## Author Contributions

FZ: provided the idea. YW: contributed to editing the manuscript. YW and XL: conceived the study, supervised the research and wrote the paper. All authors contributed to the article and approved the submitted version.

## Conflict of Interest

The authors declare that the research was conducted in the absence of any commercial or financial relationships that could be construed as a potential conflict of interest.
